# Effect of glaucoma on eye movement patterns and laboratory-based hazard detection ability

**DOI:** 10.1371/journal.pone.0178876

**Published:** 2017-06-01

**Authors:** Samantha Sze-Yee Lee, Alex A. Black, Joanne M. Wood

**Affiliations:** 1 School of Optometry and Vision Science, Queensland University of Technology, Brisbane, Queensland, Australia; 2 Institute of Biomedical and Health Innovation, Queensland University of Technology, Brisbane, Queensland, Australia; University of Leicester, UNITED KINGDOM

## Abstract

**Purpose:**

The mechanisms underlying the elevated crash rates of older drivers with glaucoma are poorly understood. A key driving skill is timely detection of hazards; however, the hazard detection ability of drivers with glaucoma has been largely unexplored. This study assessed the eye movement patterns and visual predictors of performance on a laboratory-based hazard detection task in older drivers with glaucoma.

**Methods:**

Participants included 30 older drivers with glaucoma (71±7 years; average better-eye mean deviation (MD) = −3.1±3.2 dB; average worse-eye MD = −11.9±6.2 dB) and 25 age-matched controls (72±7 years). Visual acuity, contrast sensitivity, visual fields, useful field of view (UFoV; processing speeds), and motion sensitivity were assessed. Participants completed a computerised Hazard Perception Test (HPT) while their eye movements were recorded using a desk-mounted Tobii TX300 eye-tracking system. The HPT comprises a series of real-world traffic videos recorded from the driver’s perspective; participants responded to road hazards appearing in the videos, and hazard response times were determined.

**Results:**

Participants with glaucoma exhibited an average of 0.42 seconds delay in hazard response time (p = 0.001), smaller saccades (p = 0.010), and delayed first fixation on hazards (p<0.001) compared to controls. Importantly, larger saccades were associated with faster hazard responses in the glaucoma group (p = 0.004), but not in the control group (p = 0.19). Across both groups, significant visual predictors of hazard response times included motion sensitivity, UFoV, and worse-eye MD (p<0.05).

**Conclusions:**

Older drivers with glaucoma had delayed hazard response times compared to controls, with associated changes in eye movement patterns. The association between larger saccades and faster hazard response time in the glaucoma group may represent a compensatory behaviour to facilitate improved performance.

## Introduction

Visual impairment from glaucoma can significantly impact on older adults’ ability to perform daily activities [1], with many patients reporting difficulty with reading, walking, and adapting to sudden changes in lighting [2]. Driving is particularly problematic among those glaucoma patients who still perform this activity [2, 3]. Indeed, older drivers with glaucoma have been reported to have poorer driving performance than controls under open-road conditions [4, 5] and in driving simulators [6, 7]. Studies have also reported that their five-year state-recorded [8] and self-reported [9] crash rates are up to 65% higher than their visually-normal counterparts.

Despite these reports of elevated crash rates of older drivers with glaucoma, little is known about the mechanisms underlying these associations. For example, while some studies have shown that increased severity of glaucomatous visual field loss is linked with elevated state-recorded [8, 10, 11] and self-reported [9, 12] crash rates, as well as poorer open-road driving performance [13], another failed to find a significant association between the extent of glaucomatous binocular field loss and on-road driving performance [14]. Indeed, in the latter study [14], alterations in oculomotor behaviour, particularly through increased glances towards areas of visual field loss, were found to be associated with better driving ability and safety among drivers with glaucoma. This suggests that there are other factors, such as eye movement patterns, that may be linked with driving ability in this population of drivers; however, these factors are not currently well understood.

A key aspect of driving performance and safety is hazard detection, where efficient detection allows drivers to make timely evasive manoeuvres to avoid traffic crashes. In spite of the importance of hazard detection and the elevated crash rates of older drivers with glaucoma, the hazard detection ability of this group of drivers has not yet been fully evaluated. However, there have been implicit suggestions that older drivers with glaucoma have reduced hazard detection ability. For example, in a computer-based hazard detection study, Crabb et al. [15] reported case examples of drivers with glaucoma who failed to detect traffic hazards appearing in areas of their binocular visual field loss. However, the authors did not report the frequency of these occurrences, and no statistical analyses were conducted on their hazard detection performance [15]. The suggestion of reduced hazard detection performance in glaucoma is supported by a small on-road study of 20 older drivers with glaucoma [16], which reported that the predominant reason for a driving instructor intervention in the driving assessment was failure to see and give way to pedestrians. However, assessment of drivers’ hazard detection ability is challenging during in-traffic assessments, given that the number and nature of hazards encountered will vary between participants due to inherent differences in traffic conditions. As such, there is an important gap in the literature regarding the hazard detection ability of older drivers with glaucoma.

Exploring the eye movement patterns of older drivers with glaucoma while performing driving-related tasks can provide information regarding their road scanning behaviour and visual attention towards potential hazards, as well as offer important insights into their hazard detection ability. Additionally, Kasneci et al. [14] suggested that eye movement behaviour could be an important factor associated with driving ability and safety among those with glaucoma. However, while eye movement studies have been conducted on adults with glaucoma while performing various daily activities, including reading [17, 18], watching television [19], face recognition [20], and other types of visual search tasks [21–24], few have been conducted for driving-related tasks [14, 15, 25]. Furthermore, these eye movement and driving studies among older drivers with glaucoma have often been limited by relatively small sample sizes, and the findings have been conflicting and inconclusive. For instance, older drivers with glaucoma have been observed to make more fixations and saccades per second relative to visually-normal controls in a driving simulator [25] and a laboratory-based hazard perception study [15]. Crabb et al. [15] suggested that the increased saccades per second adopted by those with glaucoma may compensate for their visual field defects, although it is unclear how these changes in eye movement patterns may be of benefit. Other studies involving older drivers with glaucoma have suggested that making larger saccades and glances towards the areas of visual field defects may improve simulator [25] and on-road driving performance [14], as well as benefit other non-driving tasks, such as face recognition [20] and visual search activities [21]. However, another driving simulator study [26] and a visual search experiment [22] found no significant difference in eye movement patterns between glaucoma and control participants, or any evidence of compensatory eye movement behaviours among those with glaucoma.

The aim of this study was to assess the laboratory-based hazard detection ability and associated eye movement patterns of older drivers with glaucoma, in comparison to controls. It was hypothesised that the older drivers with glaucoma would exhibit reduced hazard detection ability compared to age-matched controls, as reflected by delayed hazard response time and delayed first fixations on hazards. Additionally, it was expected that larger saccades would be linked with better laborarory-based hazard detection performance in the glaucoma group, given a previous report of a possible link between saccade amplitude and driving simulator performance [25]. This study also investigated the association between hazard detection ability and various measures of vision function, given that this relationship has not been previously explored.

## Methods

### Participants

Thirty older drivers with glaucoma (age 71 ± 7 years; 40% males) and 25 age-matched controls (72 ± 7 years; 41% males) were recruited from the Queensland University of Technology Optometry clinic and the laboratory’s existing database of participants. Participants with glaucoma had been diagnosed by an ophthalmologist, and were either using topical glaucoma medications or had undergone surgical or laser treatment, and had a worse-eye visual field mean deviation (MD) of ≤−4 decibels (dB) [16] as measured on the Humphrey Field Analyzer (HFA) 24–2 SITA-Standard program. Control participants were free of any ocular anomalies that might affect driving performance, as determined by the Optometry clinic records and an ocular health screening conducted prior to testing, which included slit lamp biomicroscopy and funduscopy. All participants were also free of any significant systemic conditions (self-reported) that may affect driving performance, such as heart disease and stroke [27]. Participants with cognitive impairments were also excluded, based on a score of < 24 on the Mini-Mental State Examination, which is a commonly-used screening tool of cognitive status [28]. All participants were given a full explanation of the nature of the study, and read and signed an informed consent form prior to participation. The study adhered to the tenets of the Declaration of Helsinki and was approved by the University’s Human Research Ethics Committee.

### Questionnaire and visual function assessment

Participants completed a self-administered questionnaire [29] that obtained information on their driving exposure, mileage, and crash history. Habitual binocular driving visual acuity (VA) was measured using a logMAR chart at a 4 metres (m) viewing distance, with a luminance of 100 candela per square metre (cd/m^2^), and scored letter-by-letter [30]. Binocular contrast sensitivity was measured using the Pelli-Robson contrast sensitivity chart at 1 m with a luminance of 110 cd/m^2^, and scored letter-by-letter [31]. Visual fields were assessed monocularly on the HFA (model 750, Carl Zeiss-Meditec, Dublin, CA, USA) using the SITA-Standard 24–2 threshold program. One glaucoma participant had only light-perception in the worse-eye from glaucomatous damage, visual fields were thus not testable for that eye; worse-eye MD was substituted with a value of −30 dB [32] for analytical purposes. The right and left monocular fields were used to generate an integrated visual field (IVF) for each participant, where the higher total deviation (more positive) value of the two eyes for each of the 52 corresponding field locations was used, excluding the two most nasal points [33]. The mean total deviation value of all the corresponding points was taken as the MD value for the IVF [33]. Binocular fields were additionally evaluated with the binocular Esterman test [34].

The commercially available version of the Useful Field of View (UFoV^®^; version 6.0.8, Visual Awareness Research Group, Punta Gorda, FL) was used to measure visual processing speeds for central vision, divided attention, and selective attention (subtests 1–3) [35]. Central motion sensitivity was measured using random-dot kinematograms (RDK) and drifting Gabor patches [36]. For RDK motion sensitivity, a central square patch of the dots moved in one of four directions (up, down, left, right), and participants verbally reported which direction the central dots were perceived to be moving. Gabor motion sensitivity was measured using a patch of vertical sinusoidal gratings, which drifted along the horizontal plane, and participants verbally reported whether the patch appeared to be drifting towards the left or the right. The computer-based stimuli for both motion tests were presented at a viewing distance of 3 m, with participants wearing their habitual driving optical correction. The threshold values for the dot and Gabor motion sensitivities were the minimum displacement threshold (log degree arc) and minimum drift rate (hertz [Hz]) respectively.

### Hazard Perception Test (HPT)

The HPT is a laboratory-based assessment used in driving research to evaluate hazard perception ability [15, 37–40]. The test is similar to that used in Australia and the UK for an open driver license [41–44], and poor performance on the test has been linked to elevated self-reported crash rates among older drivers [37]. The HPT comprises a series of video clips of real-world driving scenes recorded through the windshield from a driver’s perspective. Participants were instructed to view these scenes as though they were the driver, and indicate the presence of any traffic hazard by clicking on the road user involved with the computer mouse. A traffic hazard was defined as “any situation where your vehicle is on course to hit another road user, and you need to slow down, brake, or change course to avoid a crash”. Each video clip contained one primary hazard, with two or fewer secondary hazards; only responses to the primary hazard were analysed. Hazards consisted of vehicles presented in a variety of hazardous situations (e.g. merging, changing lanes, pulling out from a side road), pedestrians, and cyclists.

Each participant viewed 20 video clips of 9–25 seconds (s) in duration (mean 15.5 ± 4.6 s) presented at 30 frames per second. The scenes subtended a visual angle of 33.9°×14.8° at a viewing distance of 64 centimetres (cm). Participants wore a trial frame with lenses which corrected for the working distance and their habitual driving correction. The primary outcome measure of the HPT was the hazard response time, measured from the start of the video clip to the moment when the hazard was clicked. As the time point that a road user appears and becomes apparent as a hazard varied across the video clips, for analytical purpose, the raw response times were converted to z-scores according to each video clip relative to the whole sample. Where participants failed to respond to a hazard, the response was substituted with a z-score of +2.0 [40, 45]. The z-scores were later converted back into hazard response times (s) using the means and standard deviations (SD) of responses from all participants and video clips [37, 40, 45] to aid in the interpretation and reporting of the results. A secondary measure was the number of hazards detected.

### Eye-tracking

While participants performed the HPT, their eye movements were recorded by a Tobii TX300 eye-tracker (Tobii Technology, Danderyd, Sweden), which is a remote infrared system that samples at 300 Hz with an accuracy of 0.3–0.6° [46]. Head movements were not restricted, which allowed natural head movements and minimized participant discomfort. Prior to testing, an in-built five-point calibration procedure was performed. Eye movement measures included the time to first fixation on each hazard, fixation time on each hazard before response, number of fixations per second, average fixation duration, average saccade amplitude, and the variance in fixation positions along the horizontal and vertical planes [40]. Fixations were defined as static eye movements with gaze positions remaining within 1.6° of visual angle for at least 100 milliseconds (ms) [40, 47, 48], while saccades were defined as the eye movements between two successive fixations. Since the time of hazard appearance varied across the videos, as per hazard response time, the time to the first fixation on each hazard was converted into z-scores for analysis, then converted back into a measure of time for reporting purposes [40].

Video clip recordings with poor eye-tracking data (>50% of data missing) were excluded from the analyses, consistent with similar eye-tracking studies [15, 40, 48]. Based on this criteria, 6.1% of the 1100 video clips were excluded; the remaining had a mean of 87.6% (SD: 10.8%) of eye movement data included in the analyses, and each participant had on average 18.8 video clips (SD: 2.3) out of 20 to analyse.

### Statistical analysis

Statistical analyses were performed using SPSS version 21.0 (SPSS, Chicago, IL), with the level of significance set at p<0.05. Group differences in driving and vision function characteristics were analysed with Independent Sample t-tests. The main effects of group (glaucoma vs. controls) on HPT performance and eye movement measures were examined with linear mixed-effects models to account for missing data, with maximum likelihood estimation and random intercepts for participants [49]. Linear mixed-effects models were compared using several covariance structures. The best fit for each variable was used, as determined by the Akaike’s Information Criteria, which provides an estimation of the goodness-of-fit of the model [49]. To explore the main effects of eye movements on hazard response time, each eye movement measure was included as a covariate in separate models, and its interaction effect with group was examined. Where interaction effects were significant, the simple effects were examined.

To explore for potential visual predictors of hazard response time, each vision measure was entered as a predictor for hazard response time in separate models, which also corrected for age as a possible confounder. Given that this was an exploratory study, adjustments were not made for multiple comparisons.

## Results

As shown in Table 1, there was no group difference in self-reported driving characteristics, including years of driving experience, the number of days driven per week, weekly mileage, and crash-involvement (p > 0.05).

**Table 1:** Driving characteristics of study sample.

	Controls (n = 25)	Glaucoma (n = 30)	p-value
Driving experience (years)	51.2 ± 9.4	52.8 ± 7.2	0.46
Days driven per week	5.0 ± 2.0	5.3 ± 1.9	0.59
Weekly mileage (km)	127.1 ± 124.9	295.1 ± 630.7	0.20
Number of drivers with a history of one or more crashes (n, %)			
• In last 12 months	1 (4.0%)	2 (6.5%)	0.69
• In last 5 years	8 (32.0%)	8 (25.8%)	0.61

Continuous variables presented as mean ± SD. *p < 0.05; **p < 0.01.

Of the vision measures, the glaucoma group had significantly worse binocular contrast sensitivity, visual fields, UFoV, and motion sensitivity on all measures compared to the controls (p < 0.05; Table 2).

**Table 2:** Visual function measures of study sample.

	Controls (n = 25)	Glaucoma (n = 30)	p-value
Binocular habitual driving VA (logMAR)	−0.08 ± 0.07	−0.04 ± 0.10	0.18
Binocular contrast sensitivity (logCS)	1.94 ± 0.02	1.78 ± 0.23	<0.001**
*Visual fields*			
• Better-eye MD (dB)	−0.25 ± 1.17	−3.14 ± 3.24	<0.001*
• Worse-eye MD (dB)	−0.87 ± 1.15	−11.89 ± 6.17	<0.001**
• Integrated visual fields MD (dB)	0.43 ± 1.48	−2.71 ± 2.59	<0.001**
• Binocular Esterman efficacy score (max 100)	99.2 ± 1.6	96.1 ± 5.2	0.004**
*UFoV (Processing speeds; ms)*			
• Subtest 1: Central processing	17.6 ± 3.5	33.6 ± 41.2	0.044*
• Subtest 2: Divided attention	71.8 ± 67.4	160.7 ± 128.6	0.002**
• Subtest 3: Selective attention	251.2 ± 69.9	350.2 ± 151.0	0.003**
*Motion sensitivity*			
• RDK motion (log degree arc)	−1.96 ± 0.13	−0.77 ± 0.27	0.002**
• Drifting Gabor (Hz)	0.08 ± 0.05	0.12 ± 0.09	0.022*

Continuous variables presented as mean ± SD.

*p < 0.05;

**p < 0.01.

**Fig 1:**
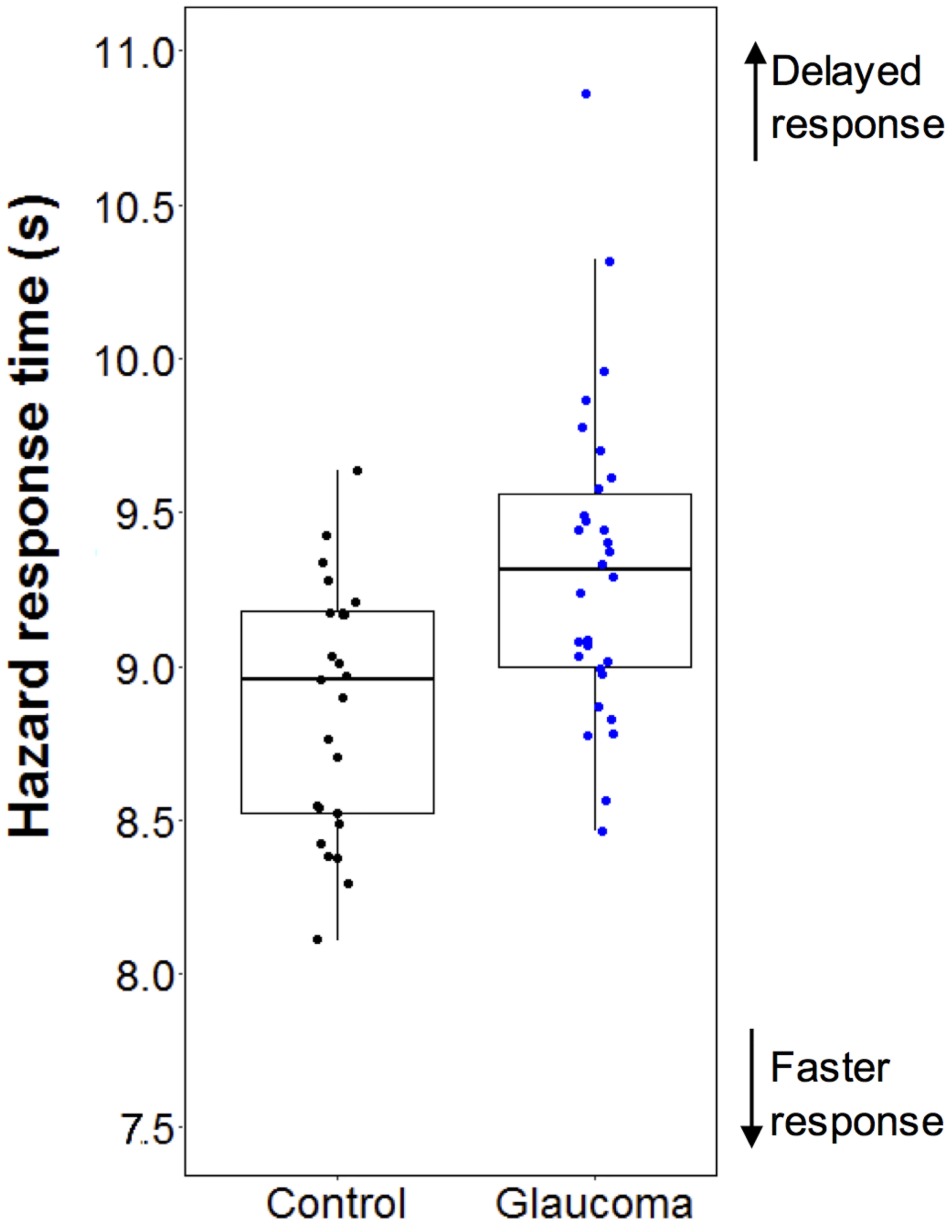
Hazard response times of the control and glaucoma group.

The glaucoma group exhibited an average of 0.42 s delay in hazard response time compared to the controls (F_1,55_ = 13.4, 95% confidence interval [CI] = 0.22−0.76 s, p = 0.001) (Fig 1 and Table 3), although the number of hazards detected did not differ significantly between groups. For the eye movements, the glaucoma group had an average of 0.30 s delay in first fixation on the hazards (F_1,45_ = 22.4, p < 0.001) and a 0.35° reduction in saccade amplitudes (F_1,56_ = 7.1, p = 0.010) compared to controls (Table 3). There was no other significant main effect of group on eye movement measures.

**Table 3:** Performance and eye movement measures of the two participant groups.

	Controls	Glaucoma	p-value
Hazard response time (s)	8.9 ± 0.9	9.3 ± 0.9	0.001**
Number of hazards detected (out of 20)	18.4 ± 2.7	18.0 ± 2.4	0.32
Time to first fixation on hazard (s)	6.3 ± 0.8	6.6 ± 1.0	<0.001**
Fixation time on hazard before response (s)	0.18 ± 0.13	0.20 ± 0.15	0.20
Number of fixations per second	1.71 ± 0.62	1.86 ± 0.61	0.14
Average fixation duration (s)	0.67 ± 0.38	0.60 ± 0.29	0.23
Average saccade amplitude (°)	4.4 ± 1.4	4.1 ± 1.3	0.010*
Horizontal search variance (°)	80.5 ± 10.5	79.3 ± 11.6	0.18
Vertical search variance (°)	32.6 ± 20.7	36.2 ± 20.8	0.07

Presented as mean ± SD.

*p < 0.05;

**p < 0.01.

There were some significant associations between eye movements and hazard response times. For all participants, faster hazard response times were associated with faster first fixations on hazards (F_1,990_ = 64.5, p < 0.001) and shorter fixation time on hazards before responding (F_1,978_ = 34.6, p < 0.001). While there was no main effect of saccade amplitude on hazard response time, there was a group × saccade amplitude interaction effect (F_1,1008_ = 9.0, p = 0.003). Simple effects analysis indicated that a 1° increase in saccade amplitude was associated with a 0.90 s faster hazard response times in the glaucoma group (F_1,546_ = 8.4, p = 0.004); however, no such relationship was found among the controls (F_1,463_ = 1.7, p = 0.19). To ensure that this association was not driven by any vision measures which may influence both the saccade amplitudes and hazard response time, additional analyses for the interaction and simple effects were conducted to correct for visual field sensitivity, UFoV, and motion sensitivity in separate models (as these were potential vision predictors of hazard response time). Even after adjustment for the vision measures, the association between saccade amplitude and hazard response time in the glaucoma group remained significant. There was no other main effect of eye movement or interaction effect with group on hazard response time.

**Fig 2:**
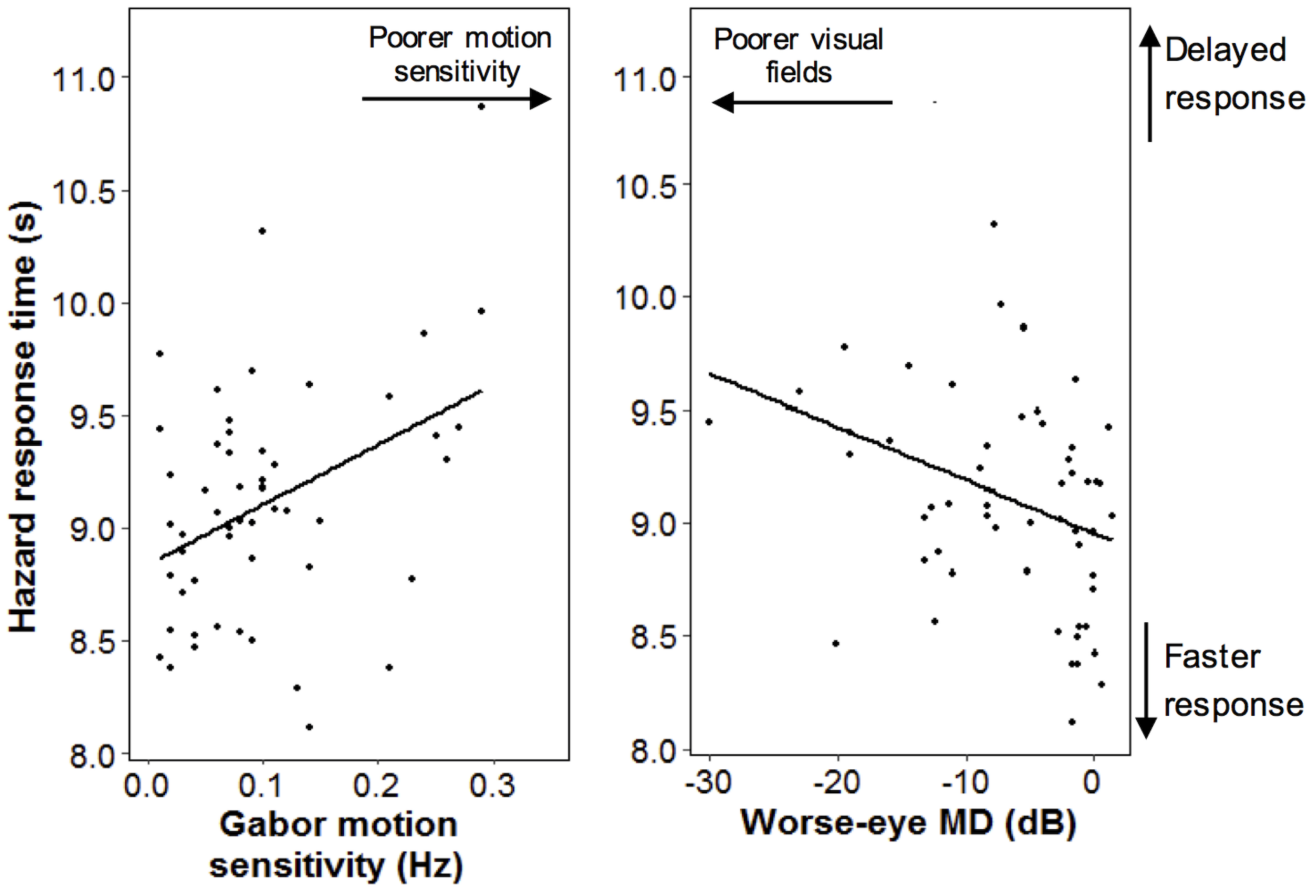
Significant associations between hazard response time and measures of visual function. Left: Gabor motion sensitivity and hazard response time; Right: worse-eye MD and hazard response times.

In age-adjusted analyses exploring the visual predictors of hazard response time for all participants, delayed hazard response time was most strongly associated with reduced Gabor motion sensitivity (F_1,53_ = 13.8, p < 0.001; Fig 2). Other significant predictors of hazard response times included worse-eye MD (F_1,55_ = 6.1, p = 0.017; Fig 2), UFoV divided and selective attention (F_1,53_ = 9.8, p = 0.003 and F_1,54_ = 5.2, p = 0.027 respectively), and RDK motion sensitivity (F_1,53_ = 9.2, p = 0.004). There were no other significant vision predictors of hazard response time.

## Discussion

This study demonstrated that older drivers with glaucoma have more delayed responses and first fixations on traffic hazards, as assessed on the HPT, compared to age-matched controls. When considered in the context of on-road driving, the glaucoma group’s 0.42 s delay in hazard response time would be equivalent to a 7 m increase in stopping distance for a vehicle travelling at 60 kilometres per hour. This delay in hazard response time may have important implications for the driving safety of older drivers with glaucoma; for example, Horswill et al. [50] reported a 0.3 s delay in hazard response time among visually-normal older drivers with a five-year history of self-reported crash involvement, compared to those who were crash-free. The current findings are supported by those of Crabb et al. [15], who observed that some glaucoma participants did not respond to hazards that appeared in the defective areas of their binocular fields in a similar HPT study, although further details and analyses of such occurrences were not provided. Among young visually-normal drivers, Glen et al. [51, 52] likewise noted that hazard detection was poorer in the presence of simulated visual field loss (simulated using a gaze-contingency paradigm) as compared to baseline (normal) viewing conditions. The authors additionally reported that defects in the superior region particularly resulted in impaired laboratory-based hazard perception ability [51]; although there was no difference in hazard detection performance for simulated defects in either the left or right regions of the visual field [52].

In addition to the delayed hazard response times, the glaucoma group exhibited more delayed first fixation on hazards than the controls. This may be because the drivers with glaucoma had more difficulty viewing the driving scenes and took longer to identify the relevant objects due to their visual field loss. These findings of delayed hazard response times and first fixations on hazard provides some evidence of impaired hazard detection ability among older drivers with glaucomatous visual impairment, which may contribute to their decreased driving performance [4, 5, 16] and increased crash rates [8, 53].

The findings also demonstrated that the glaucoma group made smaller saccades than the controls. A likely reason for the decrease in saccade amplitudes with glaucoma may be reduced visual field sensitivity or constricted functional fields, resulting in decreased awareness of peripheral objects, as suggested in studies on individuals with visual field loss from retinitis pigmentosa [54] or simulated impairments [55]. Another potential reason for the decrease in saccadic amplitudes in glaucoma is that the damage to the retinal ganglion cells indirectly leads to neurological signal deficits in the areas of the brain involved in saccadic control [56, 57]. Indeed, it has been demonstrated that patients with glaucoma, even among those without any clinically-measurable visual field defects, exhibit more saccadic errors [58, 59], including reduced saccade amplitudes and accuracy, particularly when viewing moving targets [59]. Conversely, other HPT [15] and non-driving studies [22–24] have failed to find significant reductions in saccade amplitudes among adults with glaucoma. Given these conflicting reports, further studies on the effect of glaucoma on saccade control during driving-related tasks are necessary.

Importantly, there was a significant relationship between larger saccades and faster hazard response times in the glaucoma group, but not among the controls. Similar observations were made in a recent qualitative simulator study [25], which found that drivers with glaucoma who passed the driving assessment (as determined by the German driving standards) made larger saccades than the controls, compared to those who failed. Glen et al. [20] likewise reported a significant positive correlation between saccade amplitude and performance on a face recognition task among glaucoma patients, but not the controls. These findings suggest the possibility of compensatory behaviours, such that increasing saccade amplitudes in adaptation to glaucomatous visual field loss may improve task performance. However, as the current study was cross-sectional in nature, the association between saccade amplitude and hazard response time does not prove any causal relationship. Other studies have found no evidence of compensation for visual field defects through alteration of eye movement patterns during driving in a simulator [26] or in a visual search task [21]. Given the conflicting evidence, further investigations on the link between driving-related outcomes and saccade amplitudes among older adults with glaucoma are warranted.

The current study also demonstrated associations between various measures of visual function and hazard detection ability. Of all the visual field measures, worse-eye MD was the only significant predictor of hazard response time. This is interesting given that natural viewing during driving involves both eyes, thus better-eye or binocular visual field measures would be expected to be more strongly associated with driving-related task performance than worse-eye MD. A possible explanation for this may be that the level of visual field loss in the better-eye and IVF in the glaucoma group was relatively mild. Nonetheless, previous studies have also noted that worse-eye MD has a stronger relationship with crash rates [9, 11, 53] and on-road driving performance [16] than the better-eye and IVF. It remains unclear why worse-eye MD was more predictive of driving-related task performance than other visual field measures, and further research is warranted to better understand this association.

Importantly, motion sensitivity was the strongest visual predictor of hazard response time. Given that the HPT presents video clips from the perspective of a driver in a moving vehicle, good motion sensitivity would be advantageous in detecting the hazards. Indeed, a previous HPT study found significant correlations between motion sensitivity and hazard perception among adults with normal vision [36]. Previous on-road studies involving older drivers [60, 61] and a simulator study involving young drivers [62] have also found motion sensitivity to be strongly associated with driving performance. Nonetheless, the current findings are the first to indicate that motion sensitivity plays a key role in hazard detection, a specific and important aspect of driving, among older drivers, including those with glaucoma.

Slower visual processing speeds were also associated with poorer hazard detection ability for both groups of participants. Given that drivers need to quickly and effectively extract visual information from their central and peripheral vision [35], good visual processing speeds would enable them to efficiently identify the relevant information from the irrelevant, facilitating hazard perception. This finding provides novel evidence of the importance of the UFoV in detecting hazards, which may be a linked to the strong association between UFoV and crash rates reported previously [63, 64].

The current findings should be considered in terms of the strengths and limitations of the study. The strengths include the use of an unobtrusive eye-tracker with high accuracy and allowing natural head movements, as well as the standardized testing protocol. However, a limitation is the small visual angle of the HPT video clips compared to other HPT studies [15, 38], due to the fixed monitor size of the eye-tracking system, which may have resulted in a conservative estimate of the group difference in eye movements and the associations with hazard response time. Moreover, the small visual angle of the HPT video clips did not allow for an exploration of response times to hazards appearing from more peripheral areas of the visual field, which often occur in the real world. Additionally, the HPT does not replicate the visually-complex and dynamic nature of real-world driving, given that the latter engages a larger area of the visual field with the driver physically moving through the environment. In addition, on-road driving involves physical control of the vehicle in terms of braking and acceleration, steering and lane keeping; it is nonetheless a feasible method of assessing hazard detection ability in a controlled way and has been shown to be associated with self-reported crash rates [37]. Furthermore, the participants with glaucoma in the current study had mild to moderate field defects, and thus their results may not fully generalize to the wider population of older drivers with glaucoma, some of whom may have more severe visual field defects. Despite these limitations, significant group differences in performance and eye movement measures were found. An additional point to note is that the current study focused only on older drivers; different results might have been obtained among younger drivers who may have the potential to make eye movement adaptations to visual impairments more efficiently than older drivers. Future investigations comparing how eye movement patterns may differ as a function of age and glaucomatous visual impairment should therefore be conducted.

In summary, this study provides novel experimental evidence that older drivers with glaucomatous visual impairment have reduced hazard detection ability, a critical aspect of driving safety, as reflected by their delayed hazard response time and first fixation on hazards on a computer-based HPT. The current study was also the first to demonstrate that delayed hazard response times were significantly associated with several measures of visual function, including motion sensitivity, worse-eye MD, and UFoV. The link between increased saccade amplitudes and faster hazard response time in the glaucoma group suggests that making larger saccades may be beneficial for road hazard detection ability among drivers with glaucomatous visual impairment. Further exploration regarding potential eye movement strategies that may improve hazard detection ability and on-road driving safety of this driving population is necessary.
